# Therapeutic approach to Gradenigo's syndrome: a case report

**DOI:** 10.1186/1752-1947-4-151

**Published:** 2010-05-24

**Authors:** Ilias Kantas, Anna Papadopoulou, Dimitrios G Balatsouras, Andreas Aspris, Nikolaos Marangos

**Affiliations:** 1 Centre of Otorhinolaryngology, Head and Neck and Skull Base Surgery, Euroclinic Athens, Greece; 2 Department of Otolaryngology, Tzanion General Hospital, Piraeus, Greece; 3 Nicosia General Hospital, Cyprus

## Abstract

**Introduction:**

Traditional management of Gradenigo's syndrome requires aggressive and radical surgery without any attempt to preserve hearing. Recent reports, however, describe a successful outcome after conservative surgical intervention without labyrinthectomy. A similar outcome has also been reported in patients who were only prescribed with antibiotics and did not undergo myringotomy.

**Case presentation:**

We report the case of a 24-year-old Caucasian Greek woman with Gradenigo's syndrome who was treated by draining her petrous apex via an infralabyrithine approach between her posterior semicircular canal and the jugular bulb. Her inner ear was not sacrificed during the procedure. She presented pre-operatively with ipsilateral conductive hearing loss, which recovered completely four weeks after the surgery.

**Conclusions:**

Patients with Gradenigo's syndrome may be successfully treated with a combination of long-term permanent drainage and ventilation of the apical cells with corresponding hearing preservation. This can be achieved via a combination of transmastoid, infralabyrinthine and suprajugular approaches, if such would be allowed by the anatomy of the region or if there is enough space between the posterior semicircular canal and the jugular bulb.

## Introduction

Apical petrositis was a common complication of acute mastoiditis prior to the widespread use of antibiotics. It reported occurred in 100,000 children with acute otitis media [1]. In 1907, Gradenigo described a syndrome characterized by a triad of symptoms related to apical petrositis. These symptoms include otorrhea and hearing loss, deep facial pain resulting from trigeminal involvement and abducens nerve paralysis [2]. The trigeminal ganglion and the sixth cranial nerve are separated from the bony petrous apex only by the dura mater, hence their vulnerability to inflammatory processes occurring within this region [3]. The involvement of the sixth cranial nerve is caused by the spread of inflammation through the Dorello's canal under the petroclinoid ligament [3,4]. The absence of abducens palsy, however, does not automatically exclude apical petrositis from the findings [5].

Computed tomography (CT) and magnetic resonance imaging are useful in the diagnosis and management of Gradenigo's syndrome [6]. The interpretation of imaging studies of the petrous apex, however, is complicated by normal anatomical variation in the degree of pneumatization in this region. Although 80% of the temporal bones are pneumatized, air cells extending to the petrous apex occur in only 30% of cases [3]. There are two main groups of apical cells: those around the semicircular canals and those around the cochlea [7]. The bony labyrinth forms a natural barrier to the free drainage of mucus or pus from these cells.

Although the disease has been typically managed with aggressive surgical intervention, the advent of antibiotics facilitated the conservative management of selected cases [8] and it appears that the issue of optimal treatment of the disease has yet to be settled. We report here the case of our patient with apical petrositis presenting with the typical Gradenigo's triad who was successfully treated via an infralabyrithine approach with the preservation of both her middle and the inner ear.

## Case presentation

**Figure 1:**
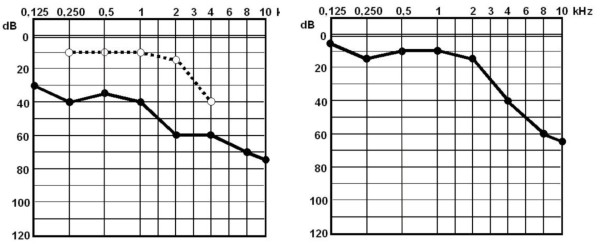
**(A). Preoperative pure tone audiogram with conductive hearing loss**. **(B) **Recovery of air conduction four weeks postoperatively (dB: Decibels; kHz: kiloHertz).

A 24-year-old Caucasian Greek woman was referred to us by her general practitioner due to the presence of acute abducens palsy a few hours prior to the referral. She had been treated with cefaclor for five days prior to her presentation because she had severe left otalgia, hearing loss and fever but no ear discharge. She was also found to have a history of infection of the upper respiratory tract one month ago. At the time of examination, she had diplopia and a complete palsy of her left sixth cranial nerve. She also complained of ipsilateral deep facial pain. Otomicroscopy subsequently revealed an acutely infected left ear with bulging tympanic membrane. A pure tone audiometry demonstrated ipsilateral conductive hearing loss (Figure 1A)

**Figure 2:**
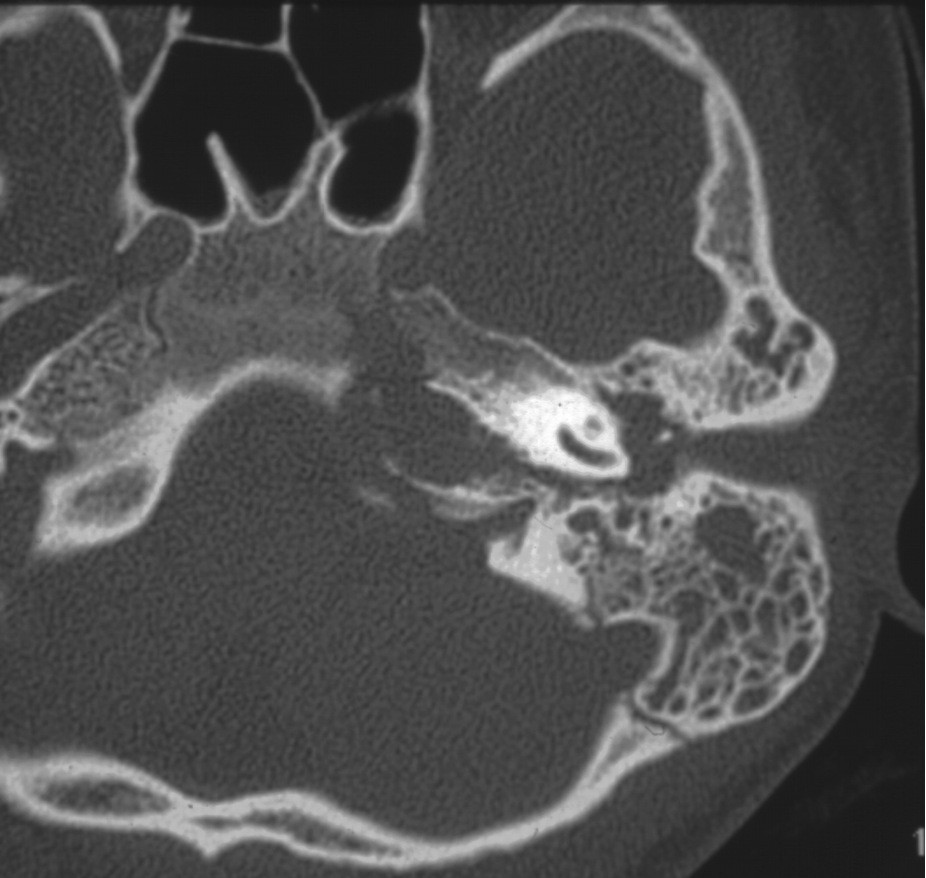
**Axial computed tomography scan demonstrating a fluid-filled mastoid cavity and a hole filled with soft tissue medially to the cochlea**. Notice the bone erosion of its walls.

Gradenigo's syndrome was initially considered, and this clinical diagnosis was confirmed by a high resolution CT of her temporal bones (Figure 2) Since the CT scan demonstrated that her jugular bulb was situated quite inferiorly under the labyrinth, our patient was scheduled for an emergency transmastoid infralabyrinthine approach. The aim of this approach was to preserve the cochleovestibular function.

A complete mastoidectomy involving the identification, without the exposure of the sigmoid sinus, was performed on our patient under general anesthesia. The mastoid segment of the facial nerve and posterior semicircular canal of our patient were also identified. Drilling was extended inferiorly and medially following the sigmoid sinus in order to expose her jugular bulb. These structures (mastoid segment of VII, posterior semicircular canal and jugular bulb) corresponded to the anterior, superior and inferior margins, respectively, of the infralabyrinthine bony dissection.

Using a diamond burr, the infralabyrinthine air cell tract was followed anteromedially between these three structures, along the long axis of the temporal bone and toward the petrous apex. This was occupied by purulent secretions under pressure, which could be drained as soon as the last bony septae was removed. The cavity was irrigated copiously with hydrogen peroxide and normal saline solution and inspected using an endoscope to ensure a complete evacuation. A large-sized, 16Ch transcutaneous silicone tube was left for two days to avoid recurrence.

**Figure 3:**
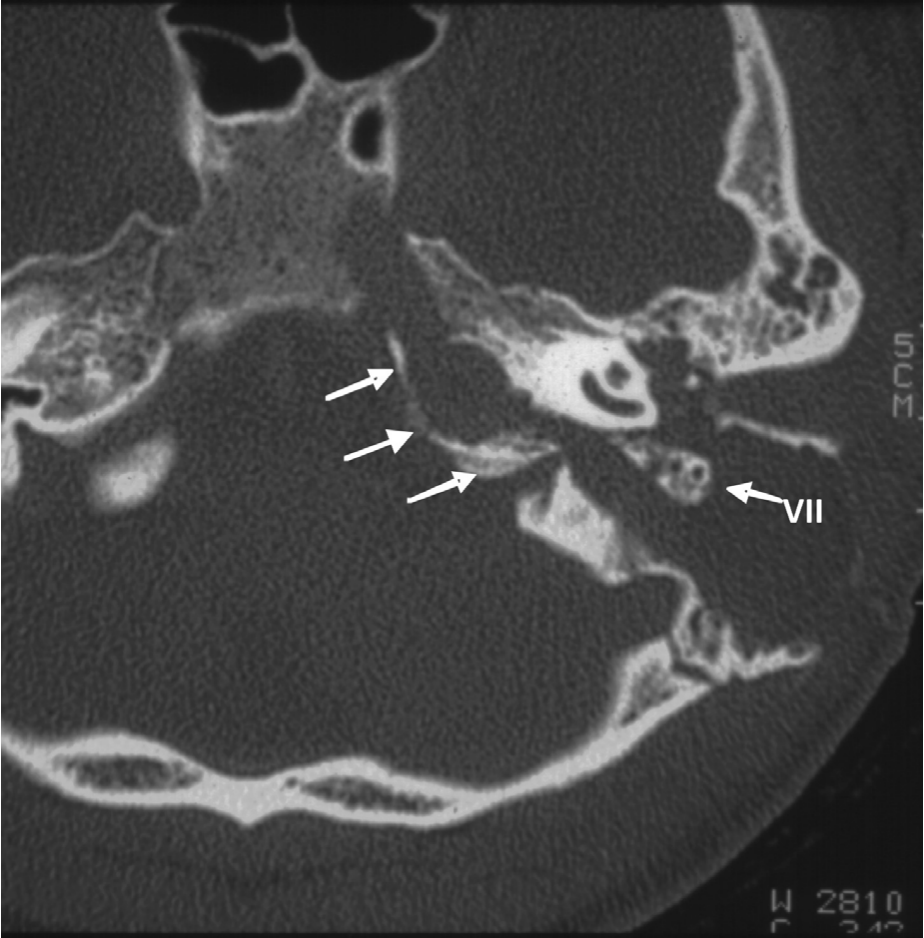
**Postoperative computed tomography scan demonstrating the canal wall-up mastoidectomy and the path to the petrous apex (arrows)**. VII indicates the mastoid portion of the facial nerve.

Cultures for aerobic and anaerobic bacteria obtained from our patient showed the presence only of *Streptococcus pneumoniae*, which was sensitive to cephalosporins. After the operation, cefuroxime was administered intravenously for the initial two days and orally for the next five days. The palsy of her sixth cranial nerve recovered completely within 24 hours. A postoperative CT scan of the temporal bones of our patient demonstrated the successful infralabyrinthine path to the petrous apex (Figure 3 ).

Four weeks after the surgery, the hematotympanum had completely resolved and the pre-operative conductive hearing loss was recovered (Figure 1B).

## Discussion

Apical petrositis has been associated with severe and life-threatening complications such as meningitis, brain abscess, lateral sinus thrombosis, or even cavernal sinus thrombosis, unless the area has been surgically decompressed and drained. Many pioneer surgeons described interventions with high morbidity and mortality and without consideration for hearing preservation [9-11]. However, Frenckner [12] described an approach through the superior semicircular canal. Eagleton [13], meanwhile, described a middle fossa approach, while Dearmin [14] and Farrior [15] described an approach between the posterior semicircular canal and the jugular bulb. All of these latter approaches attempted to preserve hearing.

The use of proper antibiotic treatment dramatically changed the incidence of the disease and its dramatic course, but surgical drainage of the petrous apex was still recommended. The management of petrous apex infection thus became more efficient. Functional preservation, especially that of hearing, then became a possibility. In more recent literature, satisfactory treatment results in patients with Gradenigo's syndrome after the administration of high doses of broad-spectrum antibiotics that penetrate the blood-cerebrospinal fluid barrier, as well as less aggressive surgery, were reported [1].

In their review of literature, Minotti and Kountakis recommended treating patients with Gradenigo's syndrome using intravenous antibiotics in conjunction with myringotomy and the insertion of large bore tympanostomy tube, unless bone erosion was evident [16]. Al-Ammar also reported a satisfactory outcome in the management of patients with Gradenigo's syndrome under conservative treatment, but still had recurrent features of the syndrome after the extrusion of the ventilation tube [4].

We believe that the management of apical petrositis should include permanent drainage and ventilation of the apical cells while also attempting to preserve hearing. This goal can be achieved through a transmastoid infralabyrinthine suprajugular approach, depending on the anatomy of the region. This means there must be enough space between the posterior semicircular canal and the jugular bulb. Careful preoperative CT evaluation, including coronal sections, is thus essential in ascertaining the applicability of this procedure. This approach allows for the exposure of all recesses that are obstructed by inflammatory lesions. It also facilitates the removal of debris, purulent secretions, septae, granulation tissue, or fibrous bands that hinder the drainage of the petrous apex. Complete drainage can be achieved by the use of sterile endoscopes.

Strategies involving myringotomy and ventilation tubes are less aggressive and may prevent the recurrence or persistence of facial palsy, but the literature provides no similar nor enough evidence pertaining to cases involving abducens palsy. Yet another unanswered question is how long the otologist should insist on conservative treatment while avoiding the deterioration of the outcome of abducens nerve palsy due to delayed surgery. It should be noted, however, that more aggressive approaches that tend to sacrifice hearing should not be totally excluded as they might be necessary whenever recurrence and life-threatening intracranial complications occur. A more aggressive approach may also be considered in cases involving pre-operative loss of conchleovestibular function and difficult anatomic configurations such as when a high jugular bulb is present.

## Conclusions

The petrous apex can be effectively drained in select cases using an infralabyrithine approach between the posterior semicircular canal and the jugular bulb, without necessarily sacrificing the function of the inner ear.

## Competing interests

The authors declare that they have no competing interests.

## Authors' contributions

IK examined and diagnosed our patient and participated in the design of the study and in drafting the manuscript. AP examined and diagnosed our patient. DB performed an audiological evaluation of our patient and helped in drafting the manuscript. GG conceived the study and examined our patient. He also reviewed the manuscript for important intellectual content. NM performed the operation on our patient and critically reviewed the manuscript. All authors read and approved the final manuscript.

## Consent

Written informed consent was obtained from our patient for publication of this case report and any accompanying images. A copy of the written consent is available for review by the Editor-in-Chief of this journal.
